# Use of ketamine in patients with refractory severe asthma exacerbations: systematic review of prospective studies

**DOI:** 10.1007/s00228-022-03374-3

**Published:** 2022-08-26

**Authors:** Luigi La Via, Filippo Sanfilippo, Giuseppe Cuttone, Veronica Dezio, Monica Falcone, Serena Brancati, Claudia Crimi, Marinella Astuto

**Affiliations:** 1 Department of Anesthesiology and Intensive Care, AOU “Policlinico – San Marco”, 95123 Catania, Italy; 2 School of Specialization in Anesthesia and Intensive Care, University of Catania, Catania, Italy; 3 School of Specialization in Anesthesia and Intensive Care, University “Magna Graecia”, Catanzaro, Italy; 4 Clinical Pharmacology Unit/Regional Pharmacovigilance Centre, University Hospital of Catania, Catania, Italy; 5 Department of Pneumology, AOU “Policlinico – San Marco”, Catania, Italy

**Keywords:** Bronchospasm, Asthma, Inflammation, Fentanyl, Aminophylline, Mechanical ventilation

## Abstract

**Purpose:**

Asthma is a heterogeneous disease with a wide range of symptoms. Severe asthma exacerbations (SAEs) are characterized by worsening symptoms and bronchospasm requiring emergency department visits. In addition to conventional strategies for SAEs (inhaled β-agonists, anticholinergics, and systemic corticosteroids), another pharmacological option is represented by ketamine. We performed a systematic review to explore the role of ketamine in refractory SAEs.

**Methods:**

We performed a systematic search on PubMed and EMBASE up to August 12th, 2021. We selected prospective studies only, and outcomes of interest were oxygenation/respiratory parameters, clinical status, need for invasive ventilation and effects on weaning.

**Results:**

We included a total of seven studies, five being randomized controlled trials (RCTs, population range 44–92 patients). The two small prospective studies (n = 10 and n = 11) did not have a control group. Four studies focused on adults, and three enrolled a pediatric population. We found a large heterogeneity regarding sample size, age and gender distribution, inclusion criteria (different severity scores, if any) and ketamine dosing (bolus and/or continuous infusion). Of the five RCTs, three compared ketamine to placebo, while one used fentanyl and the other aminophylline. The outcomes evaluated by the included studies were highly variable. Despite paucity of data and large heterogeneity, an overview of the included studies suggests absence of clear benefit produced by ketamine in patients with refractory SAE, and some signals towards side effects.

**Conclusion:**

Our systematic review does not support the use of ketamine in refractory SAE. A limited number of prospective studies with large heterogeneity was found. Well-designed multicenter RCTs are desirable.

**Supplementary Information:**

The online version contains supplementary material available at 10.1007/s00228-022-03374-3.

## Introduction

Asthma is a heterogeneous disease characterized by chronic airway inflammation and remodeling, responsible for variable airflow obstruction, thickening of the airway wall and increased mucus production. These pathophysiological features determine a wide range of symptoms such as wheezing, dyspnea, chest tightness and cough, which may vary over time in onset, frequency and intensity [1]. Asthma prevalence ranges from 1 to 21% [2] in the adult population, with a significant health and economic burden [3], of note, the incidence of asthma has increased by nearly 30% in the last 20 years [4]. Moreover, despite the availability of effective and tailored pharmacological treatments [5–8] targeting patients’ inflammatory and clinical phenotypes [9, 10], satisfactory control of asthma symptoms is still an unmet need [11] and a major challenge for clinicians [12]. Suboptimal control of asthma may lead to frequent exacerbations and admission to the emergency department for acute asthma attack. In particular, severe asthma exacerbation (SAE) is a condition characterized by a progressive increase in symptoms and with associated severe bronchospasm requiring emergency room visits, monitoring and possibly hospitalization.

First-line management of SAEs includes inhaled short-acting β-agonists, anticholinergics, and systemic corticosteroids, with the goals of relieving airflow obstruction and hypoxemia as quickly as possible; in refractory cases of SAE, intravenous magnesium sulfate and aminophylline can also be considered for the in-hospital management [13]. Noninvasive ventilatory support is often required in SAE cases [14] and nearly 10% of hospitalized SAE patients will also need intensive care unit admission. In the 2% most severe cases, intubation and invasive mechanical ventilation will also be required [15] with possible continuous infusion of muscle relaxants.

In addition to the conventional strategies for the treatment of SAE, another pharmacological option may be represented by ketamine [16, 17]. Ketamine is a rapid onset drug with well-known sedative, analgesic and antiemetic effects [18]. The use of ketamine in severe asthma has been advocated for its sympathetic stimulation and the consequent relaxation of smooth muscles and bronchodilation [19]. Therefore, ketamine may improve lung compliance and reduce airways resistances when administered as a continuous infusion. Moreover, it may increase bronchial secretions which may relieve mucus plugs [20]. Suggested dosages have been in the range of 0.5 to 2 mg/kg/h [16]. Nonetheless, ketamine has several dose-dependent side effects, such as hypertension, tachycardia, increase in intracranial pressure and sedative effects. Moreover, it can cause drooling, myoclonia, nystagmus, hallucinations and psychomotor agitation crises [18]. There are conflicting clinical reports on the value of using ketamine in patients with SAE. Therefore, we performed a systematic search of the literature to explore the role of ketamine in acute severe asthma unresponsive to conventional treatment.

## Materials and methods

### Search strategy and registration

We undertook a systematic web-based advanced literature search through the *NHS Library Evidence* tool on the effects of Ketamine in unresponsive asthma.

The protocol of our systematic review was regularly registered on PROSPERO (identified record number CRD42021273466). We followed the approach suggested by the PRISMA statement for reporting systematic reviews and meta-analyses [21] and a PRISMA checklist is provided separately (Supplementary information 1).

Our core search was structured by combining the two main terms of the topic: “*ketamine*” AND “*asthma*”. An initial computerized search of PubMed was conducted from inception until August 12^th^, 2021 to identify the relevant articles. We also performed a search on EMBASE limited to the findings from 2016 in order to retrieve the newest conference abstracts not yet published to allow a reasonable time for the peer-review process. Two further searches were performed manually and independently by three authors, also exploring the list of references of the findings of the systematic search. Inclusion criteria were pre-specified according to the PICOS approach (Table 1).

**Table 1:** PICOS Criteria

**PICOS**	
Participants	Adult and pediatric patients with severe asthma refractory to conventional therapy
Intervention	Ketamine
Comparison	Placebo or other pharmacological strategies
Outcome	Improvement in oxygenation parameters; amelioration of clinical conditions; reduction of escalation to invasive ventilation; facilitation in weaning from mechanical ventilation; decrease in peak inspiratory pressures and increase in lung compliance; evaluation of side effects
Studies included	Randomized controlled trials; prospective studies for sensitivity analysis only

After an initial decision to include all type of studies regardless of their methodological design, we preferred to select only prospective studies (randomized or not) in order to focus on higher quality and level of evidence. Regarding the population, we accepted studies focusing on both adults and pediatric patients where ketamine was used to treat refractory asthma and patients in the control group received placebo or other second-tier drugs for severe asthma. We excluded retrospective studies, case series and case reports; we also discarded experimental animal studies, book chapters, reviews, editorials and letters to the editor. Language restrictions were applied: we read the full manuscript only for articles published in English. For studies published in other languages, we read the abstract and contacted the authors for further information, if necessary. Study selection for determining the eligibility for inclusion in the systematic review and data extraction was performed independently by four reviewers. Discordances were resolved by two senior authors. Data were inserted in a password-protected Excel database.

### Outcomes analysis

We primarily compared the effects of ketamine as adjunctive therapy for severe refractory asthma on oxygenation and respiratory parameters (i.e. peak inspiratory pressures, airways resistance, lung compliance), and clinical status, need for invasive ventilation and effects on weaning from mechanical ventilation. As a secondary focus of our analysis, we evaluated the reported side effects in the patients treated with ketamine compared to the control group. We considered the possibility to perform a quantitative assessment (meta-analysis) if at least three studies consistently reported the same outcome.

### GRADE of evidence

Grade of evidence performed according to the recommendations of the Grading of Recommendations Assessment, Development and Evaluation working group was preliminarily considered only if meta-analysis was feasible.

## Results

**Fig. 1:**
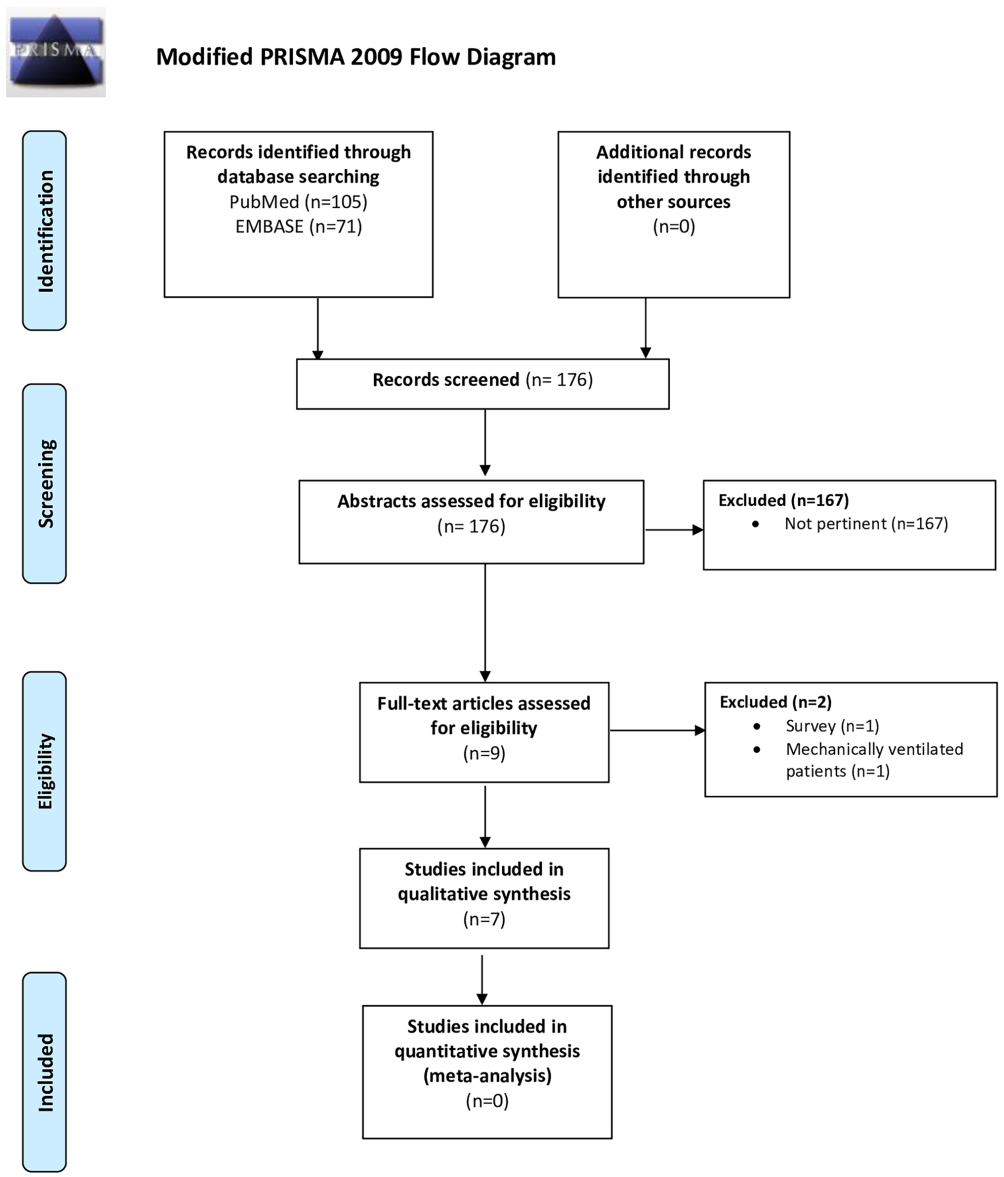
Modified PRISMA 2009 flow diagram

From our systematic search, 105 items were found on Pubmed and 71 on EMBASE (Fig. 1). We selected the potentially relevant articles and subsequently reviewed their full-text against our PICOS criteria. We initially included 9 studies, but one was subsequently excluded because it was a national survey conducted in Chile reporting the use of pharmacological and non-pharmacological approaches, outcomes and costs of the management of the asthma exacerbations in the pediatric population. Another study was excluded as after evaluation of full text it was not focused on asthma but included a heterogeneous population of mechanically ventilated patients admitted to intensive care who subsequently developed bronchospasm (defined as a thoracic compliance below 35 mL/cmH_2_O) [22].

Therefore, we included a total of 7 studies, including 5 RCTs [23–27] with a population ranging from 44 to 92 enrolled patients, and 2 prospective studies of 10 and 11 patients respectively (without the control group) [28, 29]. Of the seven included studies, four enrolled adults only [24, 26, 27, 29] and three focused on the pediatric population [23, 25, 28].

Table 2 describes the characteristics of the included studies and the main results reported by the authors. With regard to the study populations, a large heterogeneity was found regarding the number of patients included and their distribution by gender and age. Regarding the inclusion criteria of the single studies, three of them [24, 27, 29] did not clearly specify the use of scores/criteria for patients’ selection. Of the remaining four studies, one used criteria defined by the authors [26], while the remaining three used known scores for lung diseases:

**Table 2:** Summary of the included studies

**First author, year, design**	**N patients** **Median Age** (**range)**	**Inclusion criteria**	**Ketamine dose(s)** **Comparison dose**	**Outcomes studied by the authors**	**Main results of each study**
Esmailian M, 2018; RCT	N = 92 48 years (34–62)	-	- K: bolus 0.3 mg/kg (16.3%), 0.4 mg/kg (15.2%), and 0.5 mg/kg (17.4%) - Placebo	PEFR before and 1 h after treatment	- PEFR baseline K_0.3_: 346 ± 85, P: 336 ± 101 (*p* = 0.60) - PEFR 1 h after K_0.3_: 416 ± 76, P: 352 ± 101 (*p* = 0.001) No side effects reported
Allen JY, 2005; RCT	N = 68 6 years (2–10)	PIS > 8	- K: bolus 0.2 mg/kg + infusion 0.5 mg/kg/h (2 h) - Placebo	PIS at 0, 30, 60, 90, and 120 min No side effects reported	- PIS baseline K: 10 ± 1, P: 10 ± 1 (MD 0.2; 95%CI [− 0.5;0.8]) - PIS at 2 h K: 3 ± 2, P: 4 ± 1 (MD 0.4; 95%CI [− 0.4;1.3])
Tiwari A, 2016; RCT	N = 48 48 months (16–144)	PRAM ≥ 5 after 2 h of standard therapy	- K: bolus 0.5 mg/kg (20 min) + infusion 0.6 mg/kg/h (3 h) - Aminophylline: 5 mg/kg bolus (20 min) + infusion 0.9 mg/kg/h (3 h)	ΔPRAM in the first 24 h, Hypertension, Tachycardia	ΔPRAM score in the first 24 h K: 4.00 ± 1.25, A: 4.17 ± 1.68 (*p* = 0.70) No side effects reported
Nedel W, 2020; RCT	N = 45 65 years (51–79)	- Adults intubated for acute bronchospasm -Rs_max_ ≥ 12 cmH_2_O/L/s	- K: bolus 2 mg/kg + infusion 2 mg/kg/h - Fentanyl: bolus 1 mcg/kg + infusion of 1 mcg/kg/h	Rsmax, ΔPEEPi, ΔC_dyn_ at 3 h and 24 h after treatment	- Rsmax at 3 h: K: 0 ± 6, F: –3 ± 8, *p* = 0.16 - Rsmax at 24 h: K: –3 ± 17, F: –3 ± 14, *p* = 0.73 - ΔPEEPi at 3 h: K: 0 (95%CI –1;1), F: –0.5 (–8;0), *p* = 0.77 - ΔPEEPi at 24 h: K: –1 (95%CI –3;1), F: –0.5 (–5;2), *p* = 0.72 - ΔC_dyn_ at 3 h: K: 0 (95%CI –2;2), F: 0 (–2;3), *p* = 0.85 - ΔC_dyn_ at 3 h: K: 1 (95%CI –6;3), F: 0.5 (–11;3), *p* = 0.35 No side effects reported
Howton JC, 1996; RCT	N = 44 33 years (26–40)	-	- K: bolus 0.1 mg/kg + infusion at 0.5 mg/kg/h - Placebo	Respiratory rate, Borg Score, Peak flow, FEV_1_ before and after treatment	- RR before vs after K: 29 ± 7 vs 24 ± 4; P: 30 ± 10 vs 24 ± 6 - Borg Score before vs after K: 6 ± 2 vs 3 ± 1; P: 6 ± 3 vs 3 ± 2 - Peak Flow before vs after K: 139 ± 53 vs 158 ± 48, P: 124 ± 49 vs 163 ± 91 - FEV_1_ before vs after K: 0.7 ± 0.3 vs 0.9 ± 0.3; P: 0.6 ± 0.4 vs 1.0 ± 0.6 - Adverse reactions K: 17.4% (95%CI 5;39), P: 4.8% (12;24). All the above results were not significant
Petrillo TM, 2001; Prospective	N = 10 8 years (5–16)	CAS > 12	- K: bolus 1 mg/kg + infusion 0.75 mg/kg/h (1 h)	CAS and PEFR before K bolus, 10 min after K bolus, and 1 h after infusion	- CAS baseline 14 (8–21). 10 min after bolus 10 (4–12), 1 h after infusion 9 (4–12). Both results *p* < 0.001 - PEFR baseline 16 ± 10 (0–46) 10 min after bolus 47 ± 14 (0–76). 1 h after infusion: 69 ± 8 (53–95). Both results *p* < 0.05 Hallucinations n = 2, hypertension n = 1, diffuse skin flushing n = 1
Heshmati F, 2003; Prospective	N = 11 30 years (15–40)	-	- K: bolus 1 mg/kg + infusion 1 mg/kg/h (2 h)	Ppeak, PaCO_2_, PaO_2_ before K bolus, 15 min after bolus and 2 h after infusion	- Ppeak baseline 75 ± 4. 15 min after bolus 50 ± 5. 2 h after infusion 40 ± 5. Both results *p* < 0.005 - PaCO_2_ baseline 71 ± 3. 15 min after bolus 64 ± 4. 2 h after infusion 45 ± 4. Both results *p* < 0.005 - PaO_2_ baseline 63 ± 4. 15 min after bolus 75 ± 4. 2 h after infusion 92 ± 4. Both results *p* < 0.005 No side effects reported

*K* ketamine, *CAS* clinical asthma score, *Cdyn* dynamic compliance, *CI* confidence interval, *FEV1* forced expiratory volume in 1 s, *MD* mean difference, *MV* mechanical ventilation, *PEEPi* positive end expiratory pressure intrinsic, *PEFR* peak expiratory flow rate, *Ppeak* pressure peak, *PIS* pulmonary index score, *PRAM* pediatric respiratory assessment measure, *RR*, respiratory rate, *Rsmax* airway resistance, *RCT* randomized controlled trial

- the Pediatric Respiratory Assessment Measure (PRAM) score, which includes 5 parameters: suprasternal retraction, contraction of the inspiratory scalene muscles, thoracic excursion, wheezing, SpO2 [25];
- the Pulmonary Index Score (PIS), which includes respiratory rates, wheezing, inspiratory/expiratory ratio, use of accessory muscles, SpO2 [23];
- the Clinical Asthma Score (CAS), which analyzes SpO2, wheezing, inspiratory breath sounds, use of accessory muscles and neurological status [28].

Regarding ketamine dosing, the included studies used different dosages of ketamine. In particular, most of the studies used an intravenous bolus dose of ketamine followed by continuous infusion [25, 26, 28–30]. In these studies, the bolus ranged from 0.1 to 2.0 mg/kg, while the infusion was used with a variable range from 0.5 to 2.0 mg/kg/h. Only one study [24] used the ketamine bolus exclusively with dosage ranging from 0.3 mg/kg to 0.5 mg/kg. Of the five randomized studies with a control group, three compared ketamine to placebo [23, 24, 27], while the remaining two used fentanyl [26] (bolus 1 mcg/kg, followed by continuous infusion at 1 mcg/kg/h) or aminophylline [25] (slow bolus of 5 mg/kg over 20 min, followed by infusion 0.9 mg/kg/h for 3 h).

The outcomes were variable in the different studies, gas exchange (PaO_2_ and PaCO_2_) and respiratory mechanics indices (Ppeak, PEFR, FEV_1_) were mainly evaluated.

Only three studies included complications as secondary outcomes [25, 27, 28]. Tiwari et al. [25] observed hypertension in n = 2/24 patients in the ketamine group vs no one in the aminophylline group (p = 0.49), and tachycardia was noted in n = /24 and n = 21/24 in the ketamine and aminophylline groups, respectively (p = 0.49).

## Discussion

The purpose of our systematic review was to summarize the clinical evidence regarding the use of ketamine in patients with severe asthma refractory to conventional medical treatment, selecting higher-quality studies (randomized and prospective only). We found a paucity of data on the possible benefits and complications related to the use of ketamine in this patient population. Together with the reduced quality and quantity of data, we also noted a profound heterogeneity in the control group, where the treatment ranged from placebo to other drugs such as fentanyl and aminophylline. The ketamine dosages used were also largely different between studies. Furthermore, the outcomes evaluated by the included studies, were profoundly variable. Therefore, we could not conduct a quantitative analysis (meta-analysis) and the evaluation remains quite subjective.

Ketamine is a phencyclidine derivative with non-competitive antagonist effects on N-methyl-D-aspartate (NMDA) receptors. However, it may clinically have numerous other effect sites, both ion channels and receptors (i.e. L-type voltage-gated Ca2 + channels, nicotinic and muscarinic acetylcholine receptors, voltage-sensitive Na + channels, μ and δ opioid receptors, etc.). This large number of target sites for ketamine may contribute to the wide range of effects of the drug [31]. Regarding the role of ketamine in asthma, bronchodilation is supposed to be a combination of several targets: direct blockade of NMDA receptor-induced airway constriction, reduction of nitric oxide levels in pulmonary tissues (down-regulation of inducible nitric oxide synthetase activity), increase in synaptic catecholamine levels (blockade of presynaptic re-uptake), inhibition of vagal outflow, direct smooth muscle relaxation by reduction of calcium influx (L-type calcium channels), reduction of inflammation with blunted macrophage recruitment and cytokine production [32–35].

Despite this background, the results obtained from the administration of ketamine in patients with severe refractory asthma seem predominantly neutral or eventually negative. Indeed, from the qualitative analysis of the included studies it would appear that ketamine did not offer particular clinical benefits. Therefore, our systematic review does not offer significant support for the clinical use of ketamine with this indication.

The only study showing some significant benefit from ketamine was conducted by Esmailian et al. [24] on 92 adults. This study was the largest one retrieved by our systematic review and measured the Peak Expiratory Flow Rate (PEFR), evaluating the effects of increasing doses of Ketamine (0.3, 0.4 or 0.5 mg/kg as a bolus only, without continuous infusion) as compared to placebo. In this study, a significant improvement in PEFR occurred for the 0.4 and 0.5 mg/kg bolus doses; however, the authors did not perform any further measurements of respiratory function and mechanics. Furthermore, the authors excluded patients reporting side effects from ketamine treatment [24]. In another study, Nedel et al. [26] compared the effects of ketamine (2 mg/kg bolus and subsequent infusion at 2 mg/kg/h) and fentanyl administration (bolus of 1 mcg/kg and continuous infusion of 1 mcg/kg/h). Main outcomes were changes in respiratory mechanics (Airway Resistances – Rsmax; intrinsic Positive End Expiratory Pressure – PEEPi; and dynamic compliance—Cdyn) at different time-points (pre-treatment, at 3 and 24 h). In both groups, there was a decrease in Rsmax and a stability of Cdyn (albeit at severely compromised values). In this sense, the decrease in respiratory resistance over the course of 24 h in these patients was almost identical between groups (ketamine and fentanyl), thus possibly attributable to other treatment strategies (b2-agonist and steroid therapy) or eventually to similar effects of ketamine and fentanyl. Interestingly, there was a progressive increase in PEEPi in both groups at 24 h. In this sense, it is possible that in the presence of low values of Cdyn, a reduction in Rsmax with an increase in minute-volume ventilation favored air trapping and lung hyperinflation. In one pediatric study, Tiwari et al. [25] compared ketamine to aminophylline and showed similar improvements in the PRAM score and gas exchange in both groups. Furthermore, the evaluation of side effects showed a similar (and high) incidence of tachycardia, while only two patients, both in the ketamine group, had developed hypertension.

Of note, during the screening and the systematic research, among the studies analyzed we also found a national multicenter survey conducted in Chile in children with asthma exacerbation [36]. In this survey, all patients received salbutamol and 98% received systemic steroid administration. Regarding the additional rescue drug therapies to improve respiratory function, the most used medication was magnesium sulfate (6%) followed by aminophylline (0.8%) and finally by an anecdotal use of ketamine (0.5%, n = 2/396). Although conducted in a single country and limited to the pediatric population, this survey confirms that ketamine remains a drug rarely used in this setting. Notably, ketamine use is banned in some countries and undergoes special legislation for its use in many others.

In summary, from this overview of the included studies, we noted an absence of any clear and relevant benefit produced by the administration of ketamine in patients with refractory asthma, and some signals towards side effects related to its use.

However, we also found a randomized study published almost 30 years ago suggesting beneficial effects of ketamine bolus (1 mg/kg) as compared to placebo in mechanically ventilated adult patients admitted to intensive care and developing bronchospasm. In particular, the authors found improvement of gas exchange with increase in oxygenation and stable values of PaCO_2_ in the ketamine group while the oxygenation worsened and the PaCO_2_ increased in the placebo group [22]. Nonetheless, the benefits of ketamine in patients with refractory asthma seem unclear and its use should be probably reserved for well-structured experimental research setting with clear objectives and outcomes. On the other hand, performing a large randomized study may be challenging as the number of patients presenting with acute refractory asthma may not be very large.

## Limitations

Our study presents several limitations. Firstly, the number of included studies was low with a paucity of patients enrolled. Secondly, the design of the papers was not homogeneous, as we considered both randomized and non-randomized prospective clinical trials. Thirdly, the results presented by the included studies were clinically heterogeneous, and therefore a meta-analysis was not feasible. Lastly, we analyzed data from pediatric and adult patients together, possibly facing a risk of bias.

## Conclusions

Our systematic review highlights that the use of ketamine currently lacks of robust data on its role in severe or refractory asthma. Current evidence does not convincingly support its use in patients with severe asthma exacerbation refractory to conventional therapy. Well-designed multicenter randomized studies are probably needed to understand the role of ketamine in this patient’s population, although recruitment may be slow.

## Supplementary Information

Below is the link to the electronic supplementary material.

Supplementary file1 (DOCX 22 KB)

## Data Availability

On request to the corresponding author.

## Abbreviations

SAEs: Severe asthma exacerbations
RCT: Randomized controlled trial
PRAM: Pediatric respiratory assessment measure
PIS: Pulmonary index score
CAS: Clinical asthma score
NMDA: N-methyl-D-aspartate
PEFR: Peak expiratory flow rate
Rsmax: Airway Resistances
PEEPi: Intrinsic Positive End Expiratory Pressure
Cdyn: Dynamic compliance.
